# Reference values for respiratory muscle strength measured with the S‐Index Test in well‐trained athletes, e‐sports athletes and age‐matched controls

**DOI:** 10.1113/EP091938

**Published:** 2024-09-19

**Authors:** Tomasz Kowalski, Adrian Wilk, Andrzej Klusiewicz, Wojciech Pawliczek, Szczepan Wiecha, Beata Szczepańska, Jadwiga Malczewska‐Lenczowska

**Affiliations:** ^1^ Department of Physiology Institute of Sport—National Research Institute Warsaw Poland; ^2^ Department of Physical Education and Health in Biala Podlaska, Faculty in Biala Podlaska Jozef Pilsudski University of Physical Education Warsaw Poland; ^3^ Department of Nutrition Physiology Institute of Sport—National Research Institute Warsaw Poland

**Keywords:** powerbreathe, respiratory muscle strength, S‐Index Test

## Abstract

Respiratory function assessment is crucial in optimizing athletic performance, safeguarding respiratory health, and ensuring athletes can perform at their peak potential while minimizing the risk of respiratory‐related issues. The S‐Index Test is a dynamic evaluation of respiratory muscle strength. However, no comprehensive reference values regarding the S‐Index Test have been reported yet. A total of 597 participants performed the S‐Index Test. They were either well‐trained athletes (WTA), or e‐sports athletes (ESA), or age‐matched controls (AMC) groups. The differences in S‐Index Test results between sexes and for group–sex, and performance calibre tier–sex interactions were examined. The relationships between S‐Index Test results and age, anthropometric indices and training experience were assessed. Reference values for all the groups were provided. Amongst athletes, the highest values were observed in swimmers and rowers, and the lowest in figure skaters and runners. The S‐Index Test results were different for the group–sex interaction (*P* = 0.004, 151.6 ± 29.0 cmH_2_O for WTA males and 109.8 ± 21.6 cmH_2_O for WTA females, 136.7 ± 28.0 cmH_2_O for ESA males and 101.8 ± 22.0 cmH_2_O for ESA females, 128.7 ± 28.8 cmH_2_O for AMC males and 70.3 ± 24.7 cmH_2_O for AMC females) and higher in males than females (*P* < 0.001, 145.1 ± 30.5 cmH_2_O for males and 100.8 ± 27.6 cmH_2_O for females). The higher athletic level, presented as performance calibre tier, was not linked to higher respiratory muscle strength in the WTA group (*P* = 0.094). However, the Bonferroni correction revealed that except for the singular tier in females, there was a significant effect for all the other tiers and sexes (*P* < 0.001). The obtained results confirm that regardless of the level of physical activity, the anthropometric features are positively linked with respiratory muscle strength. Furthermore, age and training experience were positively correlated with the S‐Index Test results in the WTA group.

## Abstract (highlights)

- **What is the central question of this study?**
  What are the characteristics of differences in respiratory muscle strength between groups of different training status, sex and performance calibre tier?
- **What is the main finding and its importance?**
  The reference values and characteristics of differences in respiratory muscle strength were provided for different populations. The reported observations may guide athletic training and inform both health and exercise professionals.

## 1 INTRODUCTION

The role of respiratory muscles in athletic performance has been debated for decades (McConnell, 2012; Patel et al., 2012), with multiple studies presenting mixed results depending on training protocols, population characteristics and sports discipline (Kowalski et al., 2024). However, respiratory muscle strength and endurance are often described as the vital components of athletic performance, facilitating efficient oxygen delivery to working muscles during exercise (McConnell, 2011). By enhancing breathing efficiency and mitigating respiratory metaboreflex, well‐trained respiratory muscles may enable athletes to sustain higher levels of exertion for longer durations, resulting in improved endurance and overall performance (Illi et al., 2012). Furthermore, well‐trained respiratory muscles contribute to faster recovery times between training sessions or competitions by lowering the blood lactate concentration (Romer et al., 2002). Their role in maintaining proper posture and balance also may minimize the risk of injuries from prolonged sitting in e‐sports athletes (Chang et al., 2015; Emara et al., 2020) or improve the movement economy during different types of physical activity (Ferraro et al., 2019; Tong et al., 2016). The respiratory muscle function affects the adaptation to altitude and athletic performance in challenging conditions (Álvarez‐Herms et al., 2018; Hinde et al., 2020). In essence, respiratory muscle strength and endurance are essential for athletes seeking to maximize their potential and excel in their sports. Moreover, multiple studies associated respiratory muscle strength with health status in various populations (Palen et al., 2004; Shei et al., 2016; Stavrou et al., 2021) underlining its importance for monitoring and optimizing health and well‐being.

Spirometry remains the primary method to evaluate respiratory function. It has proved to be an effective tool for identifying, monitoring and managing patients dealing with lung disorders (Barreiro & Perillo, 2004). Moreover, spirometry may be crucial in optimizing athletic performance, safeguarding respiratory health, and ensuring athletes can perform at their peak potential while minimizing the risk of respiratory‐related issues (Lazovic et al., 2015; Price & Hull, 2014). Maximum inspiratory pressure (MIP) testing is often conducted alongside spirometry and contributes to assessing overall respiratory function (Laveneziana et al., 2019). MIP reflects the strength of respiratory muscles and may be applied in diagnosing, phenotyping and assessing treatment or training efficacy (Laveneziana et al., 2019). However, in many countries, spirometry testing falls under medical examination regulations, limiting its availability to specialized health professionals (Townsend, 2020). Moreover, MIP relies on static effort and is considered a quasi‐isometric measurement (Kowalski & Klusiewicz, 2023). ​​Since static evaluations were reported to fail in predicting functional muscular capacities in multiple studies (Feeler et al., 2010; Murphy & Wilson, 1996; Schapmire et al., 2010), a dynamic assessment called the S‐Index Test was recommended as a more adequate assessment of respiratory muscle strength in sports settings (Kowalski & Klusiewicz, 2023). It is noteworthy that attenuation of the respiratory metaboreflex followed changes in respiratory muscle strength (Chan et al., 2023).

The S‐Index Test, developed by POWERbreathe^®^ (POWERbreathe International Ltd, Southam, UK), is an evaluation of respiratory muscle function based on maximum dynamic inspiratory pressure. Recent studies have validated the assessment with excellent day‐to‐day reliability (Areias et al., 2020; Minahan et al., 2015). The S‐Index Test and MIP do not present similar values as they evaluate the different events of muscular contraction. However, the S‐Index Test results are strongly correlated with MIP (*r* = 0.74) (Areias et al., 2020). The test may be conducted with mobile, handheld devices, and as of today, there are no established limitations concerning the qualifications of practitioners (Kowalski et al., 2024). Therefore, the S‐Index Test is gaining popularity in sports settings as a measure of both respiratory muscle strength and endurance.

Both spirometry and MIP reference values are collected and presented to inform the decisions of health professionals (Quanjer et al., 2012; Sclauser Pessoa et al., 2014). However, to our knowledge, no comprehensive reference values regarding the S‐Index Test have been reported. Therefore, we performed multiple S‐Index Tests in the selected populations of well‐trained athletes, e‐sport athletes and age‐matched controls to establish normative reference ranges that could be used by coaches, health professionals and scientists. Moreover, we investigated the differences between the groups, sexes and performance calibre tiers. Additionally, an exploratory analysis to generate initial insights that may guide further research was performed.

## 2 METHODS

The research adhered to the *Declaration of Helsinki* guidelines. The Institute of Sport—National Research Institute Ethics Committee reviewed and approved the study protocol (approval no. KEBN‐23‐80‐TK). The participants provided written consent to take part in the study and were informed about the applied procedures in written form. They were free to withdraw at any time with no additional repercussions.

### 2.1 Participants

A total of 597 participants were recruited to participate in the study (*n* = 254 women, *n* = 343 men). The participants were either well‐trained athletes (WTA), or e‐sports athletes (ESA) or age‐matched controls (AMC). All the recruitment and testing procedures were completed between January 2023 and March 2024. The total required sample size was 281, as calculated with G*Power (version 3.1.9.2; Heinrich‐Heine‐Universität Düsseldorf, Düsseldorf, Germany), with the level of significance set at α = 0.05, power (1 − β) = 0.95, and effect size ƒ = 0.3 (ANOVA, main effects and interactions).

The WTA group consisted of 431 participants recruited from the national Olympic sports teams and professional clubs, which routinely cooperate with the Institute of Sport—National Research Institute in Warsaw, Poland. The athletes representing three different countries were invited directly by the investigators during their sojourn at the Institute. The inclusion criteria were: valid medical certificate to compete in the respective sport, semi‐professional or professional training experience of at least 2 years for Development National Teams or 4 years for Elite National Teams, and performance calibre corresponding to Highly Trained/Elite/World Class, according to the Participant Classification Framework (McKay et al., 2022). The exclusion criteria were: ongoing medication intake, any acute or chronic medical condition, smoking, or any ongoing allergy reaction. Before the assessment, all the participants from this group were examined by a physician to ensure appropriate health status. The WTA group was subsequently divided based on sports discipline. Moreover, the athletes were classified according to their sports performance calibre with the Participant Classification Framework (McKay et al., 2022), as presented in Table 1.

**TABLE 1:** Participant classification framework adopted and adapted from McKay et al. (2022).

Tier	Selected classification criteria
Tier 5: world class	Athletes who have earned Olympic or World Championship medals World record holders, as well as those achieving performances within a narrow margin of 2% of world records or exhibiting world‐leading performances Competitors ranked in the top 3−20 globally or within the top 3−10 positions at significant events like the Olympics or World Championships
Tier 4: elite/international level	Participation in international competitions, whether as individuals or as part of a national team in team sports Ranking in the top 4−300 globally, adjusted according to the scale and competitiveness of the event Attainment of performances within approximately 7% of world‐record standards or showcasing world‐leading achievements
Tier 3: highly trained/national level	Participation in national‐level competitions Achieving performances within approximately 20% of world‐record standards or demonstrating world‐leading performances Engaging in structured and periodized training programmes aimed at reaching approximately 20% of maximal or nearly maximal standards within their sport
Tier 0−2: sedentary, recreationally active and trained	Individuals with lower physical activity or sports performance than tiers 3−5

The ESA group consisted of 27 participants recruited with convenience sampling. The inclusion criteria were: age between 18 and 39 years old, at least 5 years of e‐sports training, and at least 10 h of weekly e‐sports training during the previous 3 months. The exclusion criteria were: ongoing medication intake, any acute or chronic medical condition, smoking or any ongoing allergy reaction.

The AMC group consisted of 139 participants recruited from university students. The students were invited by the investigators through announcements during on‐campus lectures and classes. The inclusion criteria were: age between 18 and 39 years old, fulfilling World Health Organization basic guidelines regarding physical activity recommending 150−300 min of moderate‐intensity aerobic physical activity, or 75−150 min of vigorous‐intensity aerobic physical activity, or an equivalent combination of moderate‐ and vigorous‐intensity activity throughout the week (Bull et al., 2020), and not training or competing professionally in any sport. The exclusion criteria were: ongoing medication intake, any acute or chronic medical condition, smoking or any ongoing allergy reaction.

The participants’ height was measured with the free‐standing digital stadiometer seca 274 (seca GmbH & Co. KG, Hamburg, Germany). The body mass was measured with the body composition analyser InBody 770 (InBodyUSA, Cerritos, CA, USA) in the WTA group and Tanita BC 418 MA (Tanita Corp., Arlington Heights, IL, USA) in the ESA and AMC groups. Body surface area (BSA) was calculated with the Mosteller Formula (Mosteller, 1987) in all groups. All the participants were Caucasian. The overall characteristics of the three groups are presented in Table 2.

**TABLE 2:** The overall characteristics of study participants.

	WTA		ESA		AMC	
Sex	Females	Males	Females	Males	Females	Males
No. of participants	192	239	5	22	57	82
Age (years)	21.5 ± 5.3	21.4 ± 5.3	21.6 ± 2.6	21.7 ± 2.1	21.6 ± 3.6	22.0 ± 1.9
Body mass (kg)	66.9 ± 23.5	77.5 ± 18.0	62.6 ± 16.5	77.3 ± 16.2	63.5 ± 10.0	81.8 ± 8.4
Body height (cm)	164.5 ± 22.7	180.0 ± 17.1	168.2 ± 8.3	183.2 ± 7.8	167.1 ± 6.7	182.4 ± 10.0
BSA (m^2^)	1.7 ± 0.2	1.9 ± 0.4	1.7 ± 0.2	2.0 ± 0.2	1.7 ± 0.2	2.0 ± 0.1

Values are mean ± standard deviation. Abbreviations: AMC, age‐matched controls; BSA, body surface area; ESA, e‐sports athletes; WTA, well‐trained athletes.

### 2.2 Testing procedure

**FIGURE 1:**
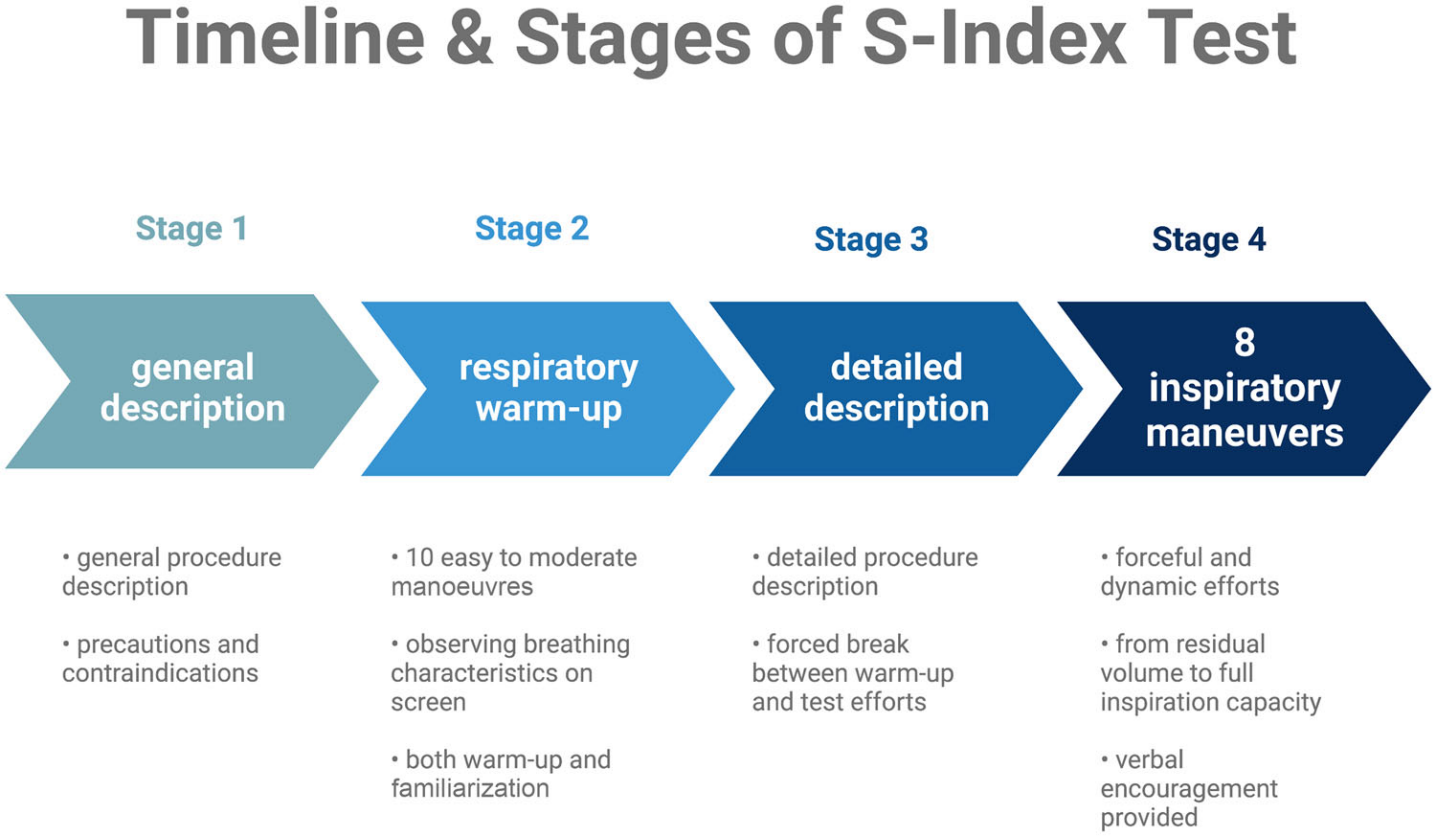
Timeline and stages of the S‐Index Test. From Kowalski and Klusiewicz (2023).

The S‐Index Tests were performed according to the guidelines presented by Kowalski and Klusiewicz (2023). The participants were advised to avoid strenuous exercise for 24 h and rested in a sitting position for 15 min before the assessment. Subsequently, they were provided with the testing procedure description and performed 10 easy inspiratory manoeuvres as a warm‐up and familiarization. Next, the participants performed eight inspiratory manoeuvres in a standing position, divided into three to four series of two to three repetitions. All the manoeuvres were executed forcefully and dynamically, from the residual volume to the vital capacity. Verbal encouragement was provided by the practitioner supervising the test. A nose clip was used to eliminate airflow outside the testing device. The testing procedure was performed with the investigators' supervision. Throughout the assessment, the participants observed their breathing characteristics in real‐time on a computer screen. A POWERbreathe K5 device with accessory software was used (POWERbreathe International Ltd, Southam, UK). The pressure and flow signals were sampled and processed at 500 Hz. The testing device was maintained and operated in line with the manufacturer's instructions. Only original disposable POWERbreathe Trysafe^®^ mouthpieces were used. The highest value obtained during one manoeuvre was presented as the S‐Index Test result (cmH_2_O). The timeline of the testing procedure is presented in Figure 1.

The WTA group's testing procedures were conducted indoors at the Institute of Sport—National Research Institute, Warsaw, Poland. The ESA and AMC group testing procedures were conducted indoors at the Jozef Pilsudski Academy of Physical Education in Warsaw, Faculty at Biala Podlaska, Poland. Both facilities are located at similar altitudes (87−139 m above sea level), longitudes and latitudes, allowing for corresponding testing conditions.

### 2.3 Statistical analysis

The results are presented as means and standard deviation. In the WTA group, for sex and sport‐specific subgroups of over 12 participants, 95% confidence intervals and quartiles were employed to characterize the data distribution and allow for convenient participant classification.

The normality of the data distribution was assessed with the Shapiro–Wilk test and visual analysis of Q‐Q plots. The assessment of differences in S‐Index Test results between sexes and for group–sex interactions was performed with analysis of variance (ANOVA) and post‐hoc Bonferroni correction if applicable. Additionally, the differences in S‐Index Test results for performance calibre tier–sex interactions in the WTA group were assessed with ANOVA and post‐hoc Bonferroni correction. Levene's test was performed to assess the variance homogeneity. Significance was set at *P* < 0.05. The effect size was determined with partial‐eta squared (η_p_^2^) and omega squared (ω^2^) (Tomczak & Tomczak, 2014). For both measures a small effect size was considered to be from 0.01 to 0.05, a medium effect size was considered to be from 0.06 to 0.14, and a large effect size was considered to be above 0.14 (Olejnik & Algina, 2003). A Spearman correlation was computed to assess the strength and direction of the relationship between S‐Index Test results and age, body height, body mass, BSA and training experience in the WTA group or age, body height, body mass and BSA in the AMC group. The statistical analyses were performed with the JASP statistical package (JASP Team, Amsterdam, Netherlands, Version 0.17.2).

## 3 RESULTS

The S‐Index Test results for WTA group divided into different sports are presented in Tables 3 and 4.

**TABLE 3:** Overall S‐Index Test results in well‐trained athletes.

Sports discipline	National team level	No of participants	PC tier	Age (years)	Training experience (years)	BSA (m^2^)	S‐Index Test result (cmH_2_O)
Females							
Overall	MX	192	3–5	21.5 ± 5.3	9.2 ± 4.9	1.7 ± 0.2	109.8 ± 21.6
Canoe/kayak	MX	26	4–5	21.9 ± 4.8	10.9 ± 5.8	1.7 ± 0.1	117.7 ± 19.9
Combat	MX	37	4–5	23.4 ± 5.3	10.5 ± 4.0	1.7 ± 0.2	106.1 ± 23.1
Cycling	MX	20	3–4	19.5 ± 3.6	7.7 ± 3.6	1.6 ± 0.4	109.5 ± 14.5
Figure skating	MX	11	3–4	18.0 ± 3.4	8.4 ± 4.8	1.5 ± 0.1	89.8 ± 18.1
Rowing	D	17	3–4	18.9 ± 0.8	3.9 ± 1.0	1.8 ± 0.1	111.1 ± 24.3
Rowing	E	13	4–5	24.2 ± 3.7	9.4 ± 3.8	1.8 ± 0.1	117.0 ± 15.3
Running	MX	9	3	18.4 ± 1.7	3.9 ± 1.5	1.6 ± 0.4	79.5 ± 19.6
Speed skating	D	12	3–4	16.6 ± 1.4	4.7 ± 1.1	1.7 ± 0.1	119.4 ± 20.0
Speed skating	E	13	4–5	23.6 ± 2.9	11.8 ± 3.3	1.6 ± 0.2	113.9 ± 13.5
Swimming	MX	18	3–5	19.9 ± 5.7	11.7 ± 3.6	1.8 ± 0.1	121.7 ± 14.0
Triathlon	MX	16	3	26.4 ± 8.6	10.4 ± 6.8	1.5 ± 0.4	111.9 ± 25.0
Males							
Overall	MX	239	3–5	21.4 ± 5.3	8.5 ± 4.7	1.9 ± 0.4	151.6 ± 29.0
Canoe/kayak	MX	13	4–5	18.3 ± 2.8	7.1 ± 1.5	1.9 ± 0.1	144.3 ± 16.2
Combat	MX	27	4–5	20.5 ± 3.1	10.3 ± 2.7	1.9 ± 0.2	141.7 ± 22.5
Cycling	MX	33	3–4	20.3 ± 6.3	7.0 ± 6.0	1.7 ± 0.6	149.9 ± 32.7
Figure skating	MX	9	3–4	20.7 ± 4.2	11.9 ± 4.2	1.8 ± 0.1	136.4 ± 14.8
Rowing	D	23	3–4	19.3 ± 0.8	5.0 ± 1.3	2.1 ± 0.1	151.6 ± 26.4
Rowing	E	16	4–5	26.5 ± 6.1	12.5 ± 5.6	2.3 ± 0.3	176.3 ± 25.7
Running	MX	23	3	22.4 ± 6.8	5.7 ± 3.9	1.8 ± 0.4	134.2 ± 20.0
Speed skating	D	24	3–4	18.4 ± 4.4	5.7 ± 2.6	1.9 ± 0.2	151.03 ± 30.3
Speed skating	E	17	4‐5	23.0 ± 1.8	11.9 ± 1.8	1.9 ± 0.2	160.3 ± 24.2
Swimming	MX	25	3–5	19.8 ± 4.1	11.3 ± 2.9	1.8 ± 0.7	172.7 ± 22.9
Triathlon	MX	29	3	22.9 ± 7.8	8.5 ± 4.9	1.8 ± 0.1	160.8 ± 32.8

Values are means ± standard deviation. The combat group includes athletes from judo, boxing, and wrestling. Abbreviations: BSA, body surface area; D, development national team; E, elite national team; F, females; M, males; MX, a mix of elite and development national team; PC, performance calibre according to the participant classification framework on a 0–5 scale (McKay et al., 2022).

**TABLE 4:** Confidence intervals and quartile distribution for S‐Index Test results in well‐trained athletes (presented only for groups of over 12 participants).

Sports discipline	National team level	PC tier	S‐Index, 95% CI (cmH_2_O)	S‐Index Q1 (cmH_2_O)	S‐Index Q2 (cmH_2_O)	S‐Index Q3 (cmH_2_O)	S‐Index Q4 (cmH_2_O)
Females							
Canoe/kayak	MX	4–5	110.0, 125.4	80.1–103.1	103.2–120.1	120.2–126.1	126.2–161.4
Combat	MX	4‐5	98.6, 113.5	53.4–87.1	87.2–111.1	111.2–121.4	121.5–151.5
Cycling	MX	3–4	103.2, 115.9	79.6–100.6	100.7–110.1	110.2–118.7	118.8–139.3
Rowing	D	3–4	99.6, 122.6	70.4–89.1	89.2–116.8	116.9–124.6	124.7–168.2
Rowing	E	4–5	108.7, 125.3	89.2–111.2	111.3–116.7	116.8–127.7	127.8–140.7
Speed skating	D	3–4	107.0, 131.8	91.6–104.2	104.3–125.8	125.9–130.8	130.9–154.7
Speed skating	E	4–5	106.6, 121.2	84.7–106.7	106.8–112.8	112.9–117.8	117.9–135.4
Swimming	MX	3–5	115.3, 128.2	88.2–119.0	119.1–123.1	123.2–128.2	128.3–142.0
Triathlon	MX	3	99.9, 123.9	72.4–94.0	94.5–109.9	110.0–120.4	120.5–169.6
MALES							
Canoe/kayak	MX	4–5	135.5, 153.1	116.4–135.0	135.1–146.1	146.2–150.0	150.1–174.0
Combat	MX	4–5	132.5, 150.9	104.0–123.6	123.7–143.2	143.3–156.5	156.6–190.6
Cycling	MX	3–4	137.8, 162.2	91.8–126.2	126.3–145.1	145.2–173.7	173.8–224.7
Rowing	D	3–4	139.0, 164.1	119.0–130.1	130.2–152.5	152.6–163.3	164.4–222.2
Rowing	E	4–5	163.3, 189.3	118.1‐159.3	159.4–172.5	172.5–194.2	194.3–221.8
Running	MX	3	125.8, 142.6	92.7–123.5	123.6–133.0	133.1–148.9	149.0–176.5
Speed skating	D	3–4	137.0, 165.1	102.2–139.8	139.9–147.5	147.5–161.0	161.1–225.8
Speed skating	E	4–5	148.4, 172.2	119.4–143.1	143.2–158.7	158.8–177.2	177.3–211.2
Swimming	MX	3–5	163.8, 181.7	136.4–159.0	159.1–175.0	175.1–188.1	188.2–230.9
Triathlon	MX	3	148.4, 173.1	107.2–136.3	136.4–154.5	154.6–181.5	181.6–234.0

The combat group includes athletes from judo, boxing and wrestling. Abbreviations: CI, confidence interval; D, development national team; E, elite national team; F, females; M, males; MX, a mix of elite and development national team; PC, performance calibre according to the participant classification framework on a 0–5 scale (McKay et al., 2022); Q1, first quartile; Q2, second quartile; Q3, third quartile; Q4, fourth quartile.

There was a statistically significant difference between S‐Index Test results and sexes (*F* = 92.82, *P* < 0.001, η_p_^2^ = 0.136, ω^2^ = 0.109). The mean values of S‐Index Test results were higher in males compared to females (145.13 ± 30.5 cmH_2_O for males and 100.78 ± 27.6 cmH_2_O for females). Moreover, there was a statistically significant effect for S‐Index Test results and the group–sex interaction (*F* = 5.48, *P* = 0.004, η_p_^2^ = 0.118, ω^2^ = 0.011). However, the Bonferroni correction revealed the lack of statistically significant effect for ESA and WTA in both sexes and ESA and AMC for both sexes. The results are presented in Table 5.

**TABLE 5:** Overall S‐Index Test results for all the well‐trained athletes, e‐sport athletes, and age‐matched controls.

Group	Sex	No. of participants	BSA (m^2^)	S‐Index Test result (cmH_2_O)
All groups	F	254	1.7 ± 0.2	100.78 ± 27.6 *†
WTA	F	192	1.7 ± 0.2	109.6 ± 21.6 *†
ESA	F	5	1.7 ± 0.2	101.8 ± 22.0 *
AMC	F	57	1.7 ± 0.2	70.3 ± 24.7 *†
All groups	M	343	1.9 ± 0.3	145.13 ± 30.5 *†
WTA	M	239	1.9 ± 0.4	151.6 ± 29.0 *†
ESA	M	22	2.0 ± 0.2	136.7 ± 28.0 *†
AMC	M	82	2.0 ± 0.1	128.7 ± 28.8 *†

Values are means ± standard deviation. *Significant differences between the sexes; †significant effects for group–sex interaction. Abbreviations: AMC, age‐matched controls; BSA, body surface area; ESA, e‐sports athletes; F, females; M, males; WTA, well‐trained athletes.

Although there was no statistically significant difference between S‐Index Test results for performance calibre tier–sex interaction in the WTA group (*P* = 0.094), the S‐Index Test results were higher in the higher tiers for both sexes, as presented in Table 6. It is noteworthy that the Bonferroni correction revealed that except for Tier 4 in females, there was a significant effect (*P* < 0.001) for all the other performance calibre tiers and sexes.

**TABLE 6:** The S‐Index Test results in well‐trained athletes for different tiers and sexes.

PC tier	Sex	No. of participants	S‐Index Test result (cmH_2_O)
5	F	44	124.3 ± 16.9
4	F	70	109.3 ± 21.5
3	F	78	102.1 ± 20.2
5	M	43	175.2 ± 23.0
4	M	86	157.6 ± 28.2
3	M	110	141.3 ± 25.3

Values are means ± standard deviation. Abbreviations: F, females; M, males; PC, performance calibre according to the participant classification framework on a 0–5 scale (McKay et al., 2022).

### 3.1 Correlations between the monitored indices

In WTA there was a positive correlation between S‐Index Test result and age (*r*(437) = 0.22, *P* ≤ 0.001), body height (*r*(437) = 0.28, *P* ≤ 0.001), body mass (*r*(437) = 0.36, *P* < 0.001), BSA (*r*(437) = 0.37, *P* < 0.001) and training experience (*r*(437) = 0.25, *P* < 0.001).

In ESA there was a positive correlation between S‐Index Test result and body mass (*r*(25) = 0.56, *P* = 0.002) and BSA (*r*(25) = 0.57, *P* = 0.002).

In AMC there was a positive correlation between S‐index test result and age (*r*(137) = 0.19, *P* = 0.022), body mass (*r*(137) = 0.60, *P* < 0.001), body height (*r*(137) = 0.61, *P* < 0.001) and BSA (*r*(137) = 0.50, *P* < 0.001).

## 4 DISCUSSION

We have presented the first comprehensive reference values regarding the S‐Index Test in different populations: well‐trained athletes, e‐sports athletes and age‐matched controls fulfilling the World Health Organization's basic guidelines for physical activity. Significantly higher S‐Index Test results were observed in males compared to females for all the groups. There was a significant difference between the S‐Index Test results for group–sex interaction. The WTA group achieved the highest values, and the AMC group achieved the lowest values. This is the first study investigating the association of athletes’ performance calibre with the S‐Index Test result in a large population of elite athletes. Noteworthy, but not statistically significant differences, were observed between the S‐Index Test results for performance tier–sex interaction. Significant associations between anthropometric indices and S‐Index Test results were found in all the groups. Furthermore, age and training experience were positively correlated with S‐Index Test results in the WTA group.

The results regarding higher S‐Index Test values in males compared to females (see Table 5) align with long‐standing observation of similar relationships in spirometry and MIP values (Klusiewicz, 2008; Quanjer et al., 2012; Sclauser Pessoa et al., 2014). This is no surprise, given that women generally have a smaller lung capacity, less diffusion surface area, lower maximum expiratory flow rates, and narrower airway diameter compared to men of similar height and age. Consequently, they demonstrate increased work of breathing, higher airway hyper‐responsiveness, and potentially greater exercise‐induced arterial hypoxaemia compared to men (Harms & Rosenkranz, 2008; Sheel et al., 2004). Therefore, the respiratory system may limit athletic performance in women more than in men (Amann, 2012), providing an important rationale for introducing RMT. It is noteworthy that RMT may be associated with higher fatigue in females than in males (Kowalski et al., 2023). Females exhibit enhanced cardiovascular reaction and more frequent symptoms such as headaches and dizziness after RMT training sessions (Kowalski et al., 2023). Interestingly, the literature reports that differences in respiratory muscle strength between sexes are significant even with allometric scaling and considering the subjects’ anthropometric variables (Chen & Kuo, 1989).

The differences in S‐Index Test results between the groups reflect the difference in traditional spirometry and static inspiratory strength measures between athletic and sedentary or recreationally active populations (Campoi et al., 2019; Vedala et al., 2013). Notably, the ESA group results were placed between the other groups. Research on ESA fitness and physiology remains scarce (Frączek et al., 2024; Sadowska et al., 2023; Seffah et al., 2023), but typically presents an average or below‐average level of cardiorespiratory fitness in ESA. The review by Seffah et al. (2023) indicates that e‐sports activities may positively impact cardiorespiratory health, but not at the level of traditional sports (Seffah et al., 2023). Our results align with these findings in the young adult population.

The S‐Index Test results were higher in higher performance tiers (see Table 6), but the differences were not statistically significant (*P* = 0.094). However, the Bonferroni correction revealed that except for Tier 4 in females, there was a significant effect (*P* < 0.001) for all the other performance calibre tiers and sexes. Overall, the link between performance calibre tier and respiratory muscle strength remains inconclusive. Practically, there is a remarkable distinction between clinical measures of performance and real‐world athletic performance in the context of worthwhile change or difference. The first may require differences larger than 10%, whereas differences below 1% are often decisive for winning medals (Currell & Jeukendrup, 2008). Although the increased respiratory muscle strength coming from RMT was proven to be a useful ergogenic training aid in multiple settings (Illi et al., 2012; McConnell, 2012), no research on respiratory muscle strength's impact on performance in large and trained populations was found by the authors of the study. Given the extraordinary demands and specific adaptations of ventilatory efficiency in athletes (Kasiak et al., 2024), our findings do not provide a clear answer regarding the respiratory system's role in athletic performance. Speculatively, the role of respiratory muscle strength may differ between the sports. Such an effect might have influenced the obtained results, as athletes' performance in different disciplines may be influenced by a distinctive set of physiological determinants with variant contribution from respiratory muscle strength.

Multiple positive associations between anthropometric indices and S‐Index Test results were found in all the groups. Moreover, in both WTA and AMC groups, a correlation between the test results and age was found. Similar findings were presented by Souza et al. (2022) in a healthy Brazilian population, who reported a significant correlation between age, body mass and height and the S‐Index Test results (Souza et al., 2022). Importantly, the observation regarding the positive association of the test results with age originates from research on healthy and young adult samples. For example, in our study, the mean age in all groups varied from 21.4 to 22.0 years. Different findings were reported in older adults and the elderly, where respiratory muscle strength and function were negatively correlated with age (Chen & Kuo, 1989; Roldán et al., 2021). Respiratory muscle strength typically begins to decrease between 40 and 60 years of age in both males and females (Sclauser Pessoa et al., 2014). However, even in the elderly the body mass and height were strongly correlated with respiratory muscle strength measured with the S‐Index Test (Roldán et al., 2021).

### 4.1 Study strengths, limitations and recommendations for further research

The strengths of the presented investigation include novelty and a large, specialized sample size of well‐trained Olympic sports and e‐sports athletes. A noteworthy limitation of the presented work is the small sample size of female ESA. All the study participants were Caucasian, and therefore our results may have limited usefulness for other races. However, the application of cross‐sectional spirometry reference equations that include race or ethnicity remains a topic of fierce, ongoing debate (Braun, 2021; Elmaleh‐Sachs et al., 2022) Additionally, applying two different body mass measurement devices may be considered a study limitation. Nevertheless, both devices have a good agreement (Kutáč & Kopecký, 2015) and may be applied in epidemiological studies (Guedes et al., 2019).

Future investigation may include other sports disciplines or thoroughly analyse the differences between different sports. Considering significant correlations between anthropometric indices and the S‐Index Test results, we recommend utilizing allometric scaling to improve the understanding of the nature of dynamic inspiratory strength. Moreover, based on differences in pre‐ and post‐exercise measurements, S‐Index testing may be applied in assessing respiratory muscle endurance. Presenting validated methodology and reference values for respiratory muscle endurance assessment might be a valuable insight for health and sports professionals. Finally, the S‐Index Test reference values may be complemented with reference equations.

### 4.2 Conclusions

Reference values for the S‐Index Test in the WTA, ESA and AMC groups have been provided. Among athletes, the highest values were observed in swimmers and rowers, and the lowest in figure skaters and runners. Regardless of sex, the athletes exhibited significantly higher respiratory muscle strength than non‐training peers, and males showed significantly higher S‐index Test values than females. The link between performance calibre tier and respiratory muscle strength in the WTA group remains inconclusive. The obtained results confirm that regardless of the level of physical activity, the anthropometric features are positively linked with respiratory muscle strength. Furthermore, age and training experience were positively correlated with the S‐Index Test results in the WTA group.

## AUTHOR CONTRIBUTIONS

Tomasz Kowalski contributed to the conception and design of the work, acquisition, analysis, and interpretation of the data, and drafted the manuscript. Adrian Wilk contributed to the acquisition of the data and drafted the manuscript. Andrzej Klusiewicz contributed to the conception and design of the work, interpretation of the data, and drafted the manuscript. Szczepan Wiecha contributed to the conception and design of the work, and interpretation of the data, and drafted the manuscript. Wojciech Pawliczek contributed to the acquisition of data for the work and revised the manuscript critically for important intellectual content. Beata Szczepańska contributed to the acquisition and analysis of the data and revised the manuscript critically for important intellectual content. Jadwiga Malczewska‐Lenczowska contributed to the conception and design of the work, and interpretation of the data, and drafted the manuscript. All authors have read and approved the final version of this manuscript and agree to be accountable for all aspects of the work in ensuring that questions related to the accuracy or integrity of any part of the work are appropriately investigated and resolved. All persons designated as authors qualify for authorship, and all those who qualify for authorship are listed.

## CONFLICT OF INTEREST

None declared.

## Data Availability

Data will be made available upon reasonable request to the corresponding author (T.K.).
